# Effect of Precursors and Their Regulators on the Biosynthesis of Antibiotics in *Actinomycetes*

**DOI:** 10.3390/molecules29051132

**Published:** 2024-03-03

**Authors:** Xu Yan, Yao Dong, Yawen Gu, Hao Cui

**Affiliations:** 1 College of Chemistry and Pharmaceutical Engineering, Jilin Institute of Chemical Technology, Jilin 132022, China; 13836954868@163.com; 2 College of Biology & Food Engineering, Jilin Institute of Chemical Technology, Jilin 132022, China; dy6224760@163.com; 3 Analytical and Testing Center of Hebei Province, Hebei University of Science and Technology, Shijiazhuang 050018, China

**Keywords:** precursor, antibiotic biosynthesis, TetR family regulators, polyketide, Actinomycetes

## Abstract

During the life activities of microorganisms, a variety of secondary metabolites are produced, including antimicrobials and antitumor drugs, which are widely used in clinical practice. In addition to exploring new antibiotics, this makes it one of the research priorities of *Actinomycetes* to effectively increase the yield of antibiotics in production strains by various means. Most antibiotic-producing strains have a variety of functional regulatory factors that regulate their growth, development, and secondary metabolite biosynthesis processes. Through the study of precursor substances in antibiotic biosynthesis, researchers have revealed the precursor biosynthesis process and the mechanism by which precursor synthesis regulators affect the biosynthesis of secondary metabolites, which can be used to obtain engineered strains with high antibiotic production. This paper summarizes the supply of antibiotic biosynthesis precursors and the progress of research on the role of regulators in the process of precursors in biosynthesis. This lays the foundation for the establishment of effective breeding methods to improve antibiotic yields through the manipulation of precursor synthesis genes and related regulators.

## 1. Introduction

Microorganisms are widely distributed in the environment, mainly in the form of spores or mycelium in the soil. *Actinomycetes* include *Streptomyces*, *Micromonospora*, *Nocardiopsis* and *Bacillus*. Many structurally novel and highly active compounds are produced during their secondary metabolism including widely used compounds such as antibiotics. *Actinomycetes* secondary metabolites, with no obvious physiological function or non-essential to the growth and reproduction of microorganisms, are produced at specific stages of growth and development generally and generated from primary metabolites as precursors. Precursors play an important role in the biosynthesis of secondary metabolites. Through the study of precursors, the mechanism of secondary metabolism biosynthesis was continuing to understand, it also provides strong support for the research and development of new antibiotic drugs. At present, two-thirds of the natural antibiotics used in clinical practice are derived from *Actinomycetes*, which contributes a lot to medical treatment. Further, *Actinomycetes* metabolites are also used in agriculture, food and other industries. However, the development and utilization of microbial secondary metabolites have been insufficient so far. The lower yield of some microbial secondary metabolites has hindered their industrial production, isolation and purification, which cannot satisfy the large demand of the market. Therefore, increasing the yield of valuable target metabolites through studies of the supply of precursors, regulatory mechanisms in the precursor biosynthetic pathway, and optimization of the biosynthetic pathway has become a hot topic for researchers.

## 2. Secondary Metabolite of *Actinomycetes*

*Actinomycetes* are the main source of natural antibiotics and 90% of them are produced by *Streptomyces*, a genus that is closely related to drug discovery and development, including antimicrobial, anticancer and immunosuppressive drug. Meanwhile, *Streptomyces* is one of the species with the highest GC content, with complex morphological differentiation and with various secondary metabolic biosynthetic pathways [1].

### 2.1. Secondary Metabolite and Biosynthesis Pathway in Actinomycetes

**Figure 1:**
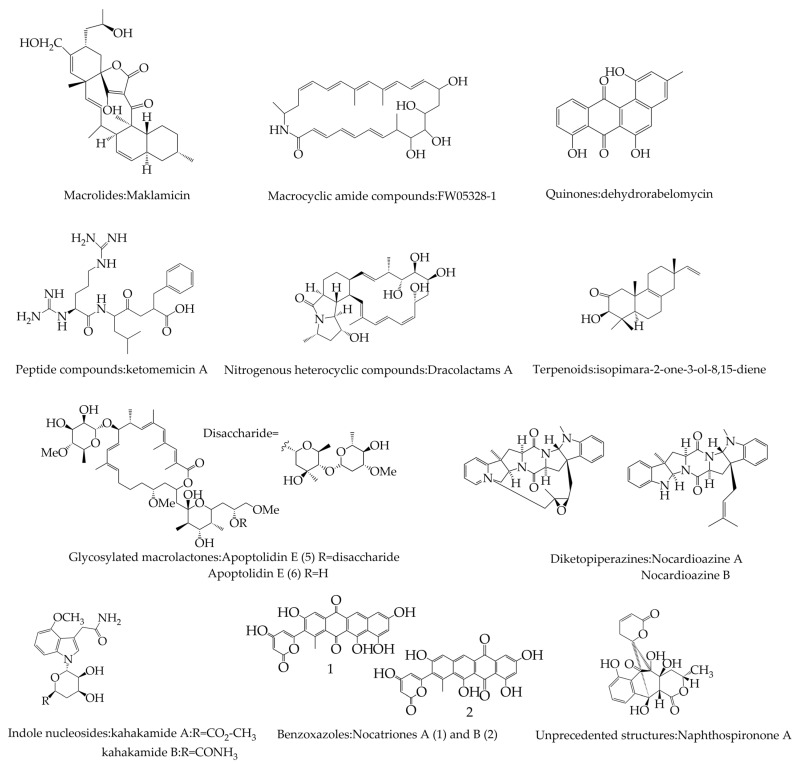
Examples of secondary metabolites in *Actinomycetes* classified according to structure.

Various secondary metabolites are produced in different species of microorganisms, which may accumulate inside the cell or be excreted into the external environment. The study of the secondary metabolism synthesis pathway of *Actinomycetes* and the exploration of the secondary metabolism regulation mechanism are important for strain breeding and generation of new antibiotics. According to the chemical structure of the products, they can be divided into antibiotics, such as aminoglycosides, macrolides, enediyne macrolides, phenazines, indoles and carbazoles, pyronidazoles and other secondary metabolites (Figure 1), as well as cellulose, degradable starch, proteins, xylan and other biomolecules with cold-, alkali- or heat-resistant enzymes with special properties. These metabolites have initially shown a broad application potential in the fields of medicine, food, biology, and chemical industry.

**Figure 2:**
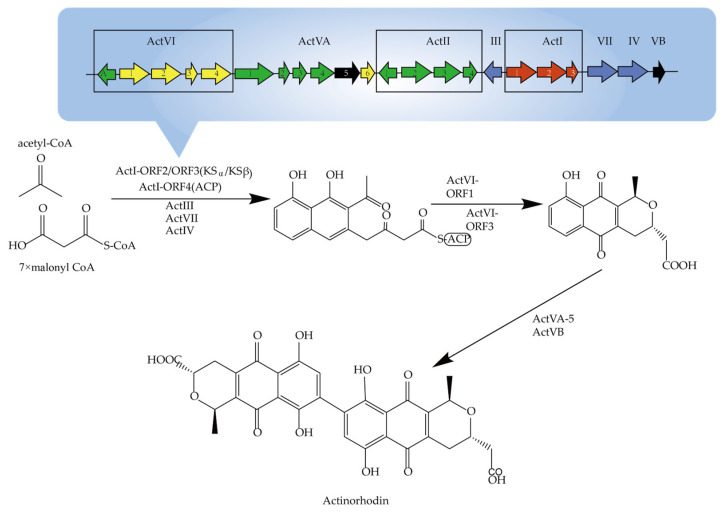
The biosynthetic gene cluster and the biosynthetic pathway of antinorhodin.

Genome sequencing revealed that biosynthetic genes in the complete pathway from primary metabolic synthesis intermediates to secondary metabolic end-products in *Actinomycetes* are usually tightly clustered. As shown in Figure 2, the earliest reported antinorhodin, produced by *S. coelicolor* and *S. lividans*, is a polyketide. Malpartida et al. achieved the first heterologous expression of a complete biosynthetic gene cluster of antibiotics in a non-antinorhodi-producing *S. parvulus*. Through the study of secondary metabolic pathways, the effective regulation of the initiation of complete pathways were discovered, as well as the horizontal transfer of genes from different sources that had occurred [2].

The biosynthetic pathway of natural products is an important research foundation in synthetic biology of natural products, and it mainly include the following pathways: the acetate-malonate pathway (AA-MA pathway), which mainly generates fatty acid analogs, phenolics, quinones, and polyketides; the mevalonate pathway (MVA pathway), which mainly produces terpenes and steroids; the shikimate pathway, which mainly generates aromatic amino acids, benzoic acids, styrenoids, phenylpropanoid-like compounds with the C6-C3 skeleton, coumarins, lignans, and lignosome; the amino acid pathway, which mainly produces alkaloids. In addition, metabolic pathways do not always exist singly, but the compounds with complex structures require different metabolic pathways to participate in the formation of metabolic networks. For example, the two intermediates of in the vincristine pathway, iridoid and tryptamine, come from the mevalonate pathway and the shikimate pathway, respectively. The more thoroughly we study the biosynthetic pathways of natural metabolites, the more favorable it will be for us to study novel metabolites.

### 2.2. Potential Secondary Metabolites of Actinomycetes

The genome of *Actinomycetes* usually contains multiple biosynthesis gene clusters of potential secondary metabolite, while most of them exist as silent clusters without corresponding products. Activation of these biosynthesis gene clusters can increase the variety of novel secondary metabolites and broaden the path of developing novel drugs. The heterologous expression is one of the ways to activate silent biosynthetic gene clusters. There are many kinds of genetic engineering methods used to isolate target biosynthetic gene clusters from the original genome, and successful expression of complex biosynthetic gene clusters have been reported. However, activation of silent gene clusters is still a difficult experimental trial-and-error process because it is difficult to predict the optimal expression host for each biosynthetic gene cluster and culture condition. Three effective methods to activate silent gene clusters can be summarized: regulatory gene modification, strong promoter introduction, and small-molecule addition. Laureti et al. identified a silent gene cluster of type I polyketide in the genome of *S. dichotomus* and activated it by overexpressing of the regulatory gene *sanmR0484*, resulting in the synthesis of the corresponding novel metabolites [3]. In addition, blocking or deleting regulators can also achieve the same effect. Christophe et al. found that deletion of the potential transcriptional repressor GbnR in *S. venezuelae* could activate the expression of the *gbn* gene cluster. Six novel γ-aminobutyric acid urease family substances (gaburedins A-F) were identified in the *gbnR* mutant strain by high-resolution LC-MS/MS [4]. The methods of modification of regulatory genes are only applicable to the secondary metabolic processes of gene clusters for which the genetic manipulation system has been established and the regulatory genes have been clearly identified, and the other silent gene clusters cannot be activated by this means alone. The use of strong promoters to activate silent gene clusters is also an effective means. Saleh et al. found that neither changing the culture conditions nor heterologous expression failed to activate the phenazine biosynthesis gene clusters in the genome of *S. tendae*. Under control of the strong promoter ermE*, the heterologous expression of the *phe* cluster were performed in *S. coelicolor* M512a, and a strong antimicrobial compound phenazine-1-carboxylic acid (PCA) as well as a new enzyme-catalyzed binding of PCA to L-type glutamine were isolated [5]. Some small molecules (antibiotics, DNA methylase and histone deacetylase inhibitors, ethanol, dimethyl sulfoxide, and heavy metal ions) are also able to affect secondary metabolic processes, causing the expression of certain silent gene clusters to produce novel secondary metabolites. For example, β-Lactams, cephalosporins and other antibiotics can act as activating factors for the silent gene clusters at low concentrations. Thus, more diversified secondary metabolites can be obtained by activating of silent gene clusters, which provides the possibility of exploring more novel drugs.

## 3. Regulation of Secondary Metabolite Synthesis in *Actinomycetes*

The metabolic process of *Actinomycetes* contains a variety of metabolic pathways and a huge regulatory system. The pathway-specific regulators play an important role in the biosynthesis of specific antibiotics, and they can also serve as a mediator for the other types of regulators to play their role in the regulation. However, global polytropic regulators not only regulate the biosynthetic pathways of various antibiotics, but also participate in the morphological differentiation of *Actinomycetes*, which is a more diversified and generalized mode of regulation compared with that of pathway-specific regulators. These regulators include TetR (Tetrepressor), LAL (Large ATP-Binding regulators of the LuxR family), SARP-LAL (*Streptomyces* Antibiotic Regulatory Proteins–LAL), PAS-LuxR, and other families, which can bind to the promoters of the target genes and affect their transcription to play the regulatory roles in the microbial metabolic processes.

### 3.1. Pathway-Specific Regulation in Secondary Metabolites

Genetic manipulation of regulatory genes is an effective method for strain improvement, and in-depth knowledge of regulatory genes is a prerequisite for enhancing antibiotic yield or activating silent gene clusters. Single or multiple pathway-specific regulatory genes that have been reported in most of the antibiotic biosynthesis gene clusters. For example, Kawachi et al. identified the pathway-specific positively regulatory gene *vmsR* in the virginiamycin pathway in *S. virginiae* [6]. Wang et al. reported that the TetR-family regulator Mfo1 affects pimaricin efflux by modulating the ATP-binding cassette (ABC) transporter Mfs1, with pimaricin and 4,5-diepoxypimaricin acting as response factors in *S. chattanoogensis* [7]. DepR1 acts as a positive regulator to promote daptomycin biosynthesis, and overexpression of *depR1* elevated daptomycin production by 41% to 474 mg/L in *S. roseosporus* [8]. Yoo et al. enhanced the yield of rapamycin to 26.7 mg/L, which was 3.7-fold higher than that of the wild strain, by knocking out the regulatory gene *rapY* in *S. rapamycinicus* [9]. Pang et al. verified the regulatory properties of the *tfpA* gene by overexpression and complementation, the results showed that the mutant strain with knockout of the *tfpA* gene increased the yield of antibiotic A21978C by 50% compared with the wild strain. In the complementary strain, the yield of antibiotic A21978C was restored to that of the wild strain, while the overexpression mutant strain almost did not produce A21978C. For this, the *tfpA* gene is a negatively regulatory gene [10].

**Figure 3:**
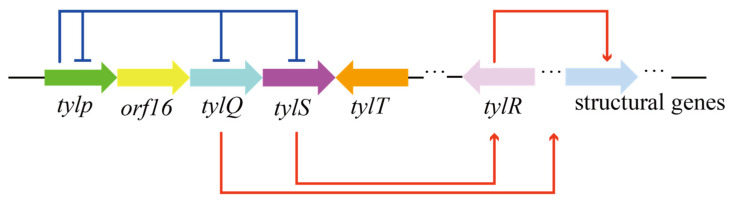
The regulatory network in the tylosin pathway formed with five TetR family regulators.

Pathway-specific regulators can also co-regulate target product synthesis by forming regulatory networks. As shown in Figure 3, there is an interacting regulatory network formed with five TetR family regulators, encoded by five pathway-specific regulatory genes, namely *tylP*, *tylQ*, *tylS*, *tylT* and *tylR,* in the tylosin pathway in *S. fradiae*. TylP, a γ-butyrolactone receptor, can repress the expression of *tylQ* and *tylS*, but also act on its own encoded genes for self-regulation. TylQ represses the production of tylosin through the modulation of the expression of the *tylR* gene. TylR is a total activator of the structural genes for the biosynthesis of tylosin [11].

### 3.2. Global Regulation in Secondary Metabolites

Most of the global regulatory genes are located outside of the biosynthesis gene cluster. Rang et al. identified a TetR-family transcriptional regulator SP_2854 in *Saccharopolyspora erythraea*, which positively regulates spinosyn biosynthesis, and affects strain growth, glucose consumption and mycelial morphology of the strain. By metabolomics analysis, it was found that overexpression of the *SP_2854* gene enhanced glucose metabolism, while deletion of the *SP_2854* gene had the opposite effect. Comparative analysis of the *SP-2854* overexpression mutant strain and the wild strain by proteomics showed that overexpression of *SP_2854* promoted the expression of glucose metabolism-related proteins, which indicated that *SP_2854* could affect the growth and development of the strain and biosynthesis of butenyl polyketide by controlling glucose metabolism [12].

The two-component system has more widespread global regulators, involved in a variety of physiological processes of strain growth, such as metabolism, virulence factor regulation, biofilm formation, and maintenance of osmotic pressure stability in *Actinomycetes*. Among the reported prokaryotic two-component regulatory systems, *absA1/A2* regulates the biosynthesis of four secondary metabolites in *S. azureus*, *cutR/S* and *afsQ1/Q2* regulate the biosynthesis of actinorhodin globally in *S. lividans* and *S. azureus*, respectively [13]. Martín-Martín et al. identified a *phoR-phoP* regulatory system which mediates the phosphate control in *S. variabilis*, in which PhoR is a receptor protein that binds to the cell membrane and PhoP binds directly to DNA. The PhoU encoding gene is linked to the PhoR-PhoP gene cluster and is expressed in the opposite direction. It was found that the *phoU*-null mutant exhibited reduced alkaline phosphatase activity, suggesting that the *PhoR-PhoP* system is required for the expression of alkaline phosphatase genes (*phoA*), and that expression of genes involved in secondary metabolic biosynthesis is regulated by the two-component PhoR-PhoP system [14]. Another type of two-component regulatory system is serine/threonine residue phosphorylation regulation. AfsK/AfsR is a group of regulatory systems in *S. azureus* and *S. griseus*. The serine/threonine protein kinase AfsK is present in the intracellular membrane and can be self-phosphorylated, whereas AfsR is a DNA-binding protein that can bind to the promoter of *afsS* to regulate the transcription of *afsS*. In *S. azureus* A3(2), KbpA, a protein encoded by the upstream gene of *afsK*, binds to the N-terminal end of non-phosphorylated AfsK, which undergoes autophosphorylation when separated by a stimulatory signal, and in turn phosphorylates AfsR to regulate secondary metabolic processes and cell differentiation [15].

**Figure 4:**
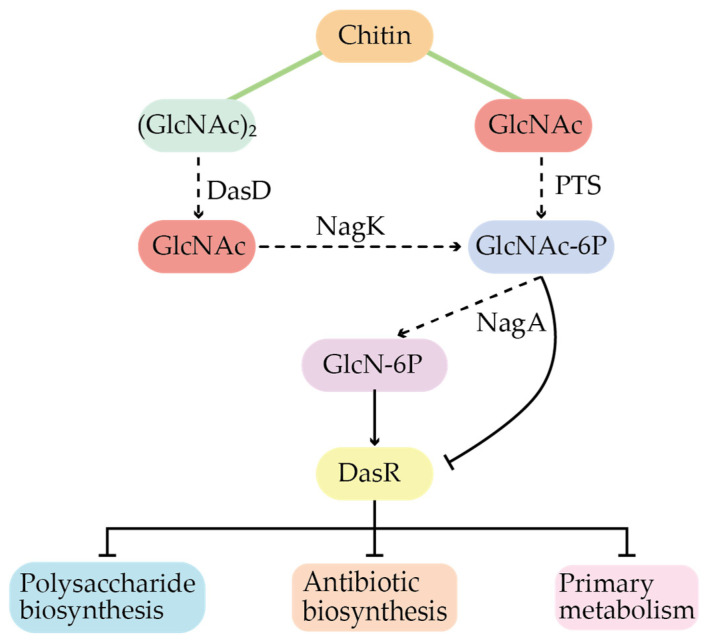
Regulatory patterns of the global regulator DasR in *S. coelicolor*.

In addition to the above two regulatory systems, there are obvious global regulatory networks in *Actinomycetes*, such as the transcriptional repressor protein DasR (first discovered and named in *S. griseus*), which has also been found in other *Actinomycetes*, such as *Bacillus subtilis* and *Saccharopolyspora erythraea*. Among them, the *S. coelicolor* is the model strain with the most relevant and systematic studies. DasR belongs to the GntR/HutC family, and a large amount of work has shown that the global regulatory protein DasR is the crossroads for balancing carbon and nitrogen source metabolism, by binding to the intermediate metabolites N-actetylglucosamine-6-phosphate (GlcNAc-6P) and Glucosamine-6-phosphate (GlcN-6P) (Figure 4) [16]. And these intermediate metabolites are often as precursors involved in the corresponding secondary metabolism, suggesting that such global regulatory proteins play an important role in mediating nutrient sensing, morphological differentiation, and primary and secondary metabolic transitions in *Streptomyces*.

## 4. Effect of Precursors on Antibiotic Biosynthesis

The biosynthesis of antibiotics requires the participation of precursors, which are mainly provided by primary metabolism. Microorganisms can introduce primary metabolites such as amino acids and acyl coenzyme A (acyl-CoA) into secondary metabolic pathways during exponential or stable phases, and produce specific compounds such as peptide antibiotics, aminoglycosides or polyketones. Sometimes, additional precursors will be synthesized to construct new secondary metabolites, such as non-protein-derived amino acids, new acyl-CoA, or alternative nucleotide-activated deoxy sugars. Therefore, secondary metabolic processes are suitable targets for modifying biosynthetic products or increasing yields by genetic engineering. Valente et al. found that *Penicillium carinaceum*, an endophytic fungus of coffee seeds, can produce mycophenolic acid and a new phthalic acid. The new products were mycophenolic acid and a new phthalic acid, 5-hydroxy-7-methoxy-4-methyl phthalic acid, mycophenolic acid and caffeine, by supplementing with the ferulic acid and quinic acid, cinnamic acid and 3,4- (methylenedioxyacid), and caffeine, respectively [17]. Wang et al. isolated three new cytochalasins from the fermentation broth of *Spicaria elegans* KLA03 by adding L-tryptophan and D-tryptophan during its cultivation [18]. Some *Actinomycetes* also change the yield of metabolites by adding precursors, and different precursors can affect the biosynthetic process and its metabolites.

### 4.1. Types of Precursors of Secondary Metabolites

Microbial secondary metabolites are commonly derived from the key intermediates of primary metabolism, which can be used as precursors for secondary metabolites [19]. Leitão classified the intermediates of primary metabolism that can be used as precursors of secondary metabolites into short-chain fatty acids, isoprenoid units, amino acids, sugars, cyclohexanols and aminocyclohexanols, squirrel radicals, purines and mithridine bases, aromatic intermediates, and methyl (-carbon pools) (Table 1) [20]. Short-chain fatty acids mainly act as precursors in the biosynthesis of polyketides and are synthesized in conjunction with carboxylase- and transferase-catalyzed reactions, condensation, and deamination reactions. Acetyl-CoA and malonyl-CoA pathways, propionyl-CoA and methylmalonyl-CoA pathways, butyryl-CoA and ethylmalonyl-CoA pathways, isobutyryl-CoA, isovaleryl-CoA, and methylbutyryl-CoA pathways and others are present in the synthesis pathway of polyketides. Isoprenoid units serve as precursor for new products by derivatizing or condensing in plants and fungi. Amino acids and modified non-protein amino acids are used as precursors in secondary metabolism through modifications such as alteration of the carbon skeleton, redox level of the molecule, cyclization to a heterocyclic ring, and racemization. Aromatic intermediates are mainly derived from the shikimate pathway. Most of the sugar precursors are derived from glucose and are incorporated in antibiotic synthesis in an overall carbon-skeleton integrated manner, which contains modification reactions by isomerization, dehydroxylation, rearrangement, decarboxylation, oxidation, and reduction. The aminocyclohexanol portion of cyclohexanol antibiotics is derived from glucose via G-6-P and myo-inositol-1P derivatization. The amidine group is mainly provided by arginine, while the methyl group is mainly derived from methionine methylation in antibiotic biosynthesis. Therefore, each class of precursor substances has similar characteristics; and during the experiment, we can use these biochemical reaction characteristics to learn their synthesis pathways, and then determine the important role of precursors in secondary metabolic pathways. In addition to the endogenous precursors in the biosynthesis process mentioned above, exogenous chemical reagents such as acetic acid and propionic acid can also be used as precursors to regulate the synthesis process. More metabolites with new structures can be obtained by changing the synthesis amount of these precursors or artificial addition.

### 4.2. Sources of Precursors for the Biosynthesis of Antibiotics

During metabolic processes, sugars can be converted to smaller five-, four-, three- and two-carbon units (such as pentose, butylose, propionate, acetate, α-ketoglutarate and oxaloacetate) through catabolic reactions. And several of these intermediates can be used as precursors for secondary metabolites directly, but some intermediates derived from glycolysis, or the tricarboxylic acid cycle need to be modified in order to serve as precursors. Some secondary metabolites of polyketide intermediates are formed from acetic acid, propionic acid, butyric acid, and other short-chain fatty acids. Among all the endogenous precursors, CoA-like precursors are one kind of the important precursors for the biosynthesis of antibiotics. Acetic acid and propionic acid, acetyl-CoA, and malonyl-CoA serve as two-carbon and three-carbon starting units, respectively, while malonyl-CoA and methylmalonyl-CoA served as normal extension units. Malonyl-CoA is formed from acetyl-CoA catalyzed by acetyl-CoA carboxylase. Propionic acid is an important precursor derived from polyketides and can be formed from succinate or isoleucine. Methyl malonyl-CoA can be formed from malonyl-CoA catalyzed by malonyl-CoA carboxylase or methylmalonic-CoA carboxyltransferase. Butyric acid is a precursor involved in several hexadecyl cyclic macrolides and polyketide antibiotics and is usually formed by condensation of an acetyl-CoA with a malonyl-CoA, or by deamination of leucine. During the biosynthesis of polyketide compounds, it is possible to regulate the synthesis of the above precursors, which in turn regulates the entire metabolic process and finally affects the yield of products.

Some fungal secondary metabolites are derived from isoprene and are involved in the biosynthesis of complex antibiotics such as neomycin. Isopentenyl pyrophosphate is formed from acetyl-CoA and mevalonate or from deamination of leucine and conversion to 3-hydroxy-3-methylglutaryl-CoA. The amino acid group of biological precursors can be used to construct isotypic peptide antibiotics or used to synthesize antibiotic precursors. There are also examples of antibiotics synthesized from intermediates in the amino acid synthesis process, such as the side chains of cephalosporins and cephalomyids, and α-aminoadipic acid (synthesized from α-ketoglutaric acid and acetyl-CoA in fungi and formed by the catabolism of lysine in bacteria). There are also many antibiotics whose aromatic portions are formed from intermediates or end-products of the manganic acid pathway. For example, the aromatic ring of actinomycin is derived from tryptophan. In contrast, sugars and amino sugars are widely present in secondary metabolites such as macrolides and aminoglycoside by the precursors of cyclohexanol [21]. This shows that secondary metabolic synthesis precursors are inseparable from primary metabolic intermediates.

### 4.3. Improved Precursor Supply Can Partially Relieve the Limitation of Product Synthesis by Insufficient Precursors

**Figure 5:**
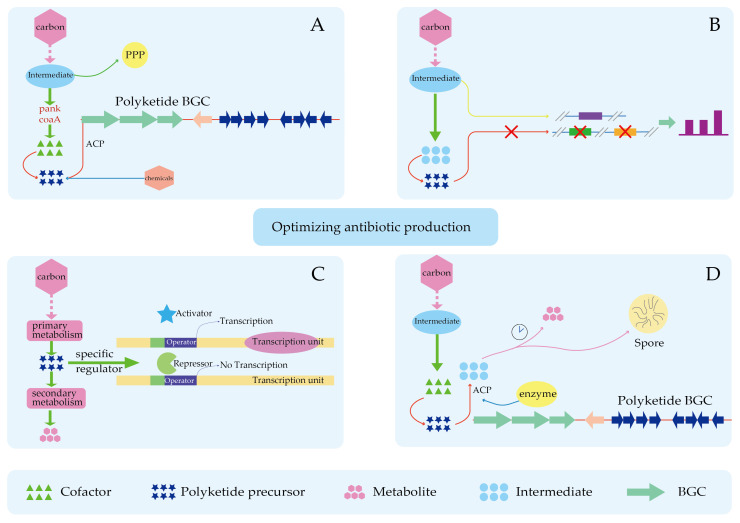
Optimizing antibiotic production. (**A**) Increase in precursor substances. (**B**) Blocking competitive pathways. (**C**) Release of inhibitory factors in precursor synthesis. (**D**) Increase precursor utilization.

The synthesis of antibiotics with insufficient precursor supply can be optimized to achieve high antibiotic yields by increasing precursor supply, blocking competitive pathways, and improving precursor utilization (Figure 5). Polyketide compounds are an important type of microbial secondary metabolites, with a wide range of biological activities, mainly formed through the successive condensation of lower carboxylic acids. Precursors are a key factor limiting the synthesis of polyketides that exist within natural strains of polyketides. Removing the limitation of precursor deficiency on polyketide synthesis is a subject of great concern. The synthesis of polyketide compounds can be promoted by improving precursor biosynthesis pathways, heterologous expression in host strains to reconstruct metabolic network, overexpression of exogenous precursor synthesis genes, inhibition of collateral metabolic flow, knockout of endogenous secondary metabolites competition pathways, and increase precursor supply. Elsayed et al. introduced an exogenous precursor pathway to promote the synthesis of their precursors and improve the yield of target products. During the fermentation of *S. natalensis*, adding 0.2% propanol directly or 2 g/L acetic acid and propionic acid mixture at a 7:1 ratio to the fermentation medium increased the yield of pimaricin by 17% or 2.5 fold, respectively [22]. The addition of glycerol, ethylene glycol and propylene glycol during the fermentation of *S. natalensis* also increased the yields of pimaricin and other polyene antibiotics, and the addition of 100 mmol/L glycerol increased the yields of nystatin, candidin and pimaricin by 0.9 fold, 2.7 fold and 2.5 fold, respectively [23]. By adding 10 mmol/L methyl oleate to the medium of *S. clavuligerus* CKD1119, the intracellular methylmalonyl-CoA concentration was 12.5-fold higher than that of the control strain, suggesting that methyl oleate may be the source of acyl-CoAs. Indirectly, the content of methylmalonyl-CoA and the yield of FK506 were increased [24].

Increasing the transcript levels of precursor biosynthetic enzymes can also increase precursor flux. The results showed that over-expressing acetyl-CoA synthetase and methylmalonyl-CoA mutase increased the yield of pimaricin by 44.19% and 20.51% in recombinant strains, respectively. Overexpression of ACS and MCM further increased the pimaricin yield of the recombinant strain (up to 1 123.34 mg/L), which was 66.29% higher than that of the wild-type strain [25]. *S. diastatochromogenes* 1628 produces guanosine triphosphate (GTP) as a precursor for nucleoside antibiotic toyocamycin (TM) with potent activity against a wide range of plant pathogenic fungi. The differentially expressed protein X0NBV6 for ribose-phosphate pyrophosphokinase (RHP) is involved in GTP biosynthesis and was expressed at a higher expression level in mutant 1628-T15, and the yield of TM produced by 1628-RHP (340.2 mg/L) was 133.3% higher than that produced by *S. diastatochromogenes* 1628 (228%) [26]. Both lincomycin and melanin use l-tyrosine as precursor, and the regulator AdpA_lin_ in *S. lincolnensis* is a pleiotropic transcriptional regulator. It regulates lincomycin precursor flux and melanin biosynthesis during biosynthesis through direct activation of *melC*, *melE* and *lmbB1/lmbB2* or inhibition of *melD* [27].

In addition to increasing precursor supply directly, it is also possible to increase precursor content and utilization indirectly by manipulating precursor synthesis genes. MatBC, a CoA thioesterase required for the conversion of exogenous malonate and methylmalonate to synthesize acyl-CoA directly in *Rhizobium tricolonoides*, was expressed heterologously in *S. azureus* resulting in a 3-fold increase in the yield of the 6-deoxy-erythromycin lactone B, which was attributed to the conversion of 1/3 of the methylmalonic acid extension unit for the synthesis of erythromycin [28]. In order to obtain more precursors, such as acetyl-CoA and malonyl-CoA, Wang et al. obtained *S. natalensis* LY08 by overexpressing the gene *ilvE*, a key enzyme for the degradation of branched-chain amino acids (BCAAs), in *S. natalensis*. The pimaricin yield of *S. natalensis* LY08 was increased by 78.72% to 1.52 g/L, which indicates that the synthesis of BCAAs can be effectively promoted by metabolic engineering to increase the supply of precursor substances [29]. Wang et al. induced the acetyl-CoA synthase encoding gene, *sco6196*, into *Streptomyces* sp. to establish a dynamic degradation system of TAG (ddTAG), which increased the acetyl-CoA content, and enhanced the yields of macrolide antibiotics, such as actinomycin, cyclase, hygromycin, and avermectin, respectively. The yield of avermectin B1a was increased by 50% from 6.20 g/L to 9.31 g/L in 180 m^3^ industrial-scale fermentation, highlighting the utility of this ddTAG strategy [30].

**Figure 6:**
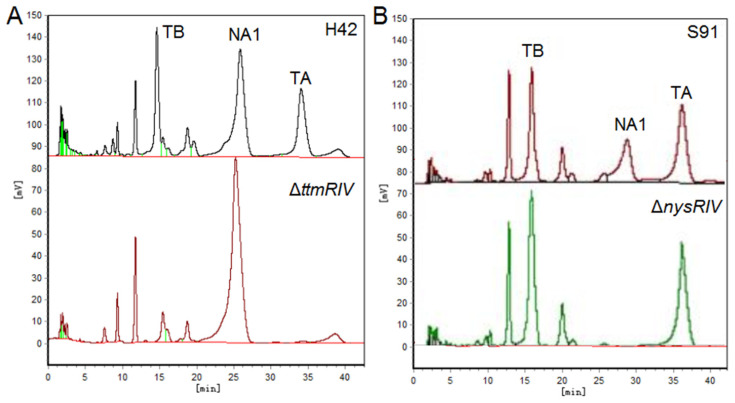
(**A**) HPLC analysis of fermentation products of H42 and Δ*ttmRIV* strain (**B**) HPLC analysis of fermentation products of S91 and Δ*nysRIV* strain. TB, tetramycin B; TA, tetramycin A; NA1, nystatin A1.

In addition, the yield of the corresponding polyene antibiotics can also be increased by blocking the biosynthetic competition pathway and altering the flow of precursors. Wuningmycin is an agricultural antibiotic for the control of fungal diseases, produced by the secondary metabolism of the *S. ahygroscopicus*, and its components include the polyene macrolides tetramycin A, tetramycin B, and nystatin. The biosynthetic pathways of tetramycin and nystatin are similar, with malonyl-CoA or methylmalonyl-CoA as the precursor to complete the polyketide skeleton assembly. As shown in Figure 6, disruption of the positively regulatory gene *ttmRIV* in the tetramycin pathway, the biosynthesis of tetramycin was blocked, and the yield of nystatin was increased, vice versa. In *S. ahygroscopicus*, deletion the tetramycin biosynthesis gene *ttmS1* completely eliminated the production of tetramycin and increased the production of nystatin by 10 fold [31]. In general, the amount of precursor synthesis could not meet the maximum level of tetramycin and nystatin synthesis. As essential substances in microbial life activities, the above precursors are strictly regulated during the synthesis process, and the metabolic balance also affects the yield of secondary metabolites.

The yield of antibiotics can also be increased by means of increased precursor utilization. The phosphopantetheinyl transferases (PPTase) are responsible for the activation of carrier proteins (acyl carrier protein, ACP or peptidyl carrier protein, PCP) in the polyketide synthases (PKS) or non-ribosomal peptide syntethases (NRPS) pathways. The expression level of PPTase can directly affect the activation efficiency of carrier proteins in PKS or NRPS. There are two PPTases, encoded by *schPPT*, responsible for ACP activation in the pimaricin pathway in *S. chattanoogensis*. Overexpression of *schPPT* accelerated the production of pimaricin and sporulation and increased the yield of pimaricin by 40% [32].

Disrupting the metabolic homeostasis of wild strains by removing inhibitory factors in precursor synthesis can also replenish the precursor supply. Kim et al. found that WblA is a global regulator with a negative regulatory role in actinorhodin biosynthesis. In addition, a TetR family transcriptional regulatory gene, *SCO1712*, was identified to be a WblA-independent actinorhodin downregulator because its disruption in *S. coelicolor* not only upregulated actinorhodin biosynthesis through pathway-specific regulators in the presence of the *wblA* transcript but also further stimulated actinorhodin production in the *wblA* deletion mutant. The *SCO5426*, a carbon flux regulating 6-phosphofructokinase gene, had no effect within the *S. coelicolor* Δ*wblA* Δ*SCO1712* mutant. Actinorhodin production was stimulated at three different levels, in *S. coelicolor* Δ*wblA* (0.10 g/g, dry weight, cells DCW at 168 h), in *S. coelicolor* Δ*wblA* Δ*SCO1712* (0.14 g/g DCW at 168 h), and in *S. coelicolor* Δ*wblA* Δ*SCO1712* Δ*SCO5426* (0.18 g/g DCW at 168 h), implying that both regulatory and precursor flux pathways could be synergistically optimized for antibiotic production [33].

## 5. TetR Family Proteins Regulate Acyl-CoA-like Precursor Synthesis via a Receptor–Ligand Model

**Figure 7:**
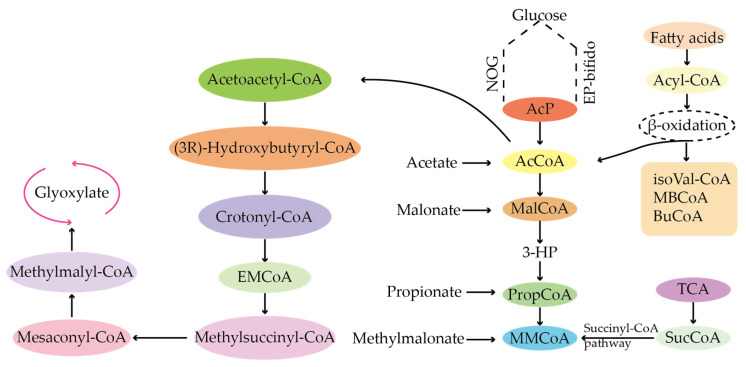
Synthesis of acyl-CoA precursors in the PKS pathway.

CoA-like substances are mainly precursor sources for the synthesis of type I PKS compounds. As shown in Figure 7, acyl-CoA-like precursor of type I polyketides includes acetyl-COA and malonyl-COA, propionyl-COA and methylmalonyl-COA, butyryl-COA and ethylmalonyl-COA, isobutyryl-COA and isovaleryl-COA, and methylbutyryl-COA [34]. TetR family regulators can regulate the synthesis process of the whole compound by regulating the synthesis of CoA analogues. Manipulation of the regulatory genes can increase the content of short-chain CoA, thereby promoting the biosynthesis of various target products. For example, malonyl-CoA is mainly produced by acetyl-CoA carboxylation catalyzed by acetyl-CoA carboxylase. The acetyl-CoA carboxylase encoding gene is the target of the TetR family regulators.

### 5.1. The TetR Family of Regulators

The TetR family of proteins is one of the most common transcriptional regulators with the role of signal transduction in *Streptomyces*. It contains 200–300 amino acid residues and contains 9–11 α-helices in the secondary structure. The first 3 to 4 helices form the Helix-Turn-Helix (HTH) structure responsible for binding to the target gene, and the remaining helices form the Ligand Binding Domain (LBD) structure responsible for binding to the ligand. In secondary metabolism of *Streptomyces*, TetR family regulators usually act as receptors and form an autoregulon-receptor regulatory system with small-molecule ligands. Ahn et al. found that most TetR family regulators were transcribed divergently from one neighboring gene in *Streptomyces*, as is the case for TetR and its target *tetA*. TetR family regulators that are 200 bp from their divergently oriented neighbors are most likely to regulate them. These target genes with various function included membrane proteins (26% of which 22% are probable membrane-associated pumps), enzymes (60%), other proteins such as transcriptional regulators (1%), and proteins having no predictive sequence motifs (13%) [35]. These results established a solid foundation for identifying targets for TetR family regulators of unknown function and demonstrated a much greater diversity of TetR family proteins regulated biochemical functions.

### 5.2. Regulation of Product Synthesis by Receptor–Ligand Pattern in the Typical Type I PKS Synthesis Pathway

**Figure 8:**
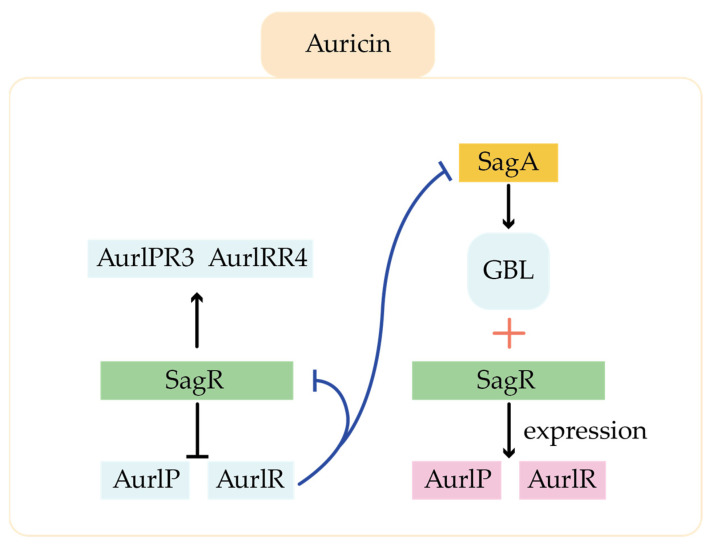
Regulation of GBL-SagA-SagR during biosynthesis of auricin.

Ligands for TetR family regulators in the type I polyketides pathway include γ-butyrolactones (GBL), self-synthesized antibiotics, 2-alkyl-4-hydroxymethylfuran-3-carboxylates, and ethanol, and the roles of this class of regulators covered a wide range of metabolic processes. For example, in the auricin pathway, there is a GBL self-regulator-receptor system (GBL-SagA-SagR), in which SagA as the GBL synthase, and SagR, a TetR family of regulator, as the GBL receptor (Figure 8). SagR can directly inhibit the transcription of pathway-specific regulatory genes *aur1P* and *aur1R*. *sagA* can be stimulated by external factors to express as GBL synthetase, which catalyzes synthesis the GBL. GBL can bind to SagR and release the *aur1P* and *aur1R* promoter regions for transcription [36]. In addition, SagR can also form a network with other regulators to play a regulatory role. Aur1P and Aur1R regulate the expression of SARP family positive regulators Aur1PR3 and Aur1PR4 to varying degrees. In addition, the negative regulator Aur1R can bind to the promoter of *sagA* and *sagR* to inhibit their transcription. SagA and SagR, negatively regulated by Aur1R, are the higher regulators in the regulatory network. In hence, several different levels of regulators form a regulatory network to precisely regulate the synthesis of auricin. Since the synthesis of secondary metabolites is closely related to the growth stage of the mycelium, the production time of auricin was significantly advanced in the *sagR*-blocked strains, suggesting that SagR also influences the morphological differentiation of the strain.

The GBL autoregulator-receptor system (GBL-SngA-SngR) is present in the pimaricin-producing *S. natalensis*. The *sngA* encodes SngA, which is involved in GBL biosynthesis, while *sngR* encodes the GBL receptor protein. Overexpression of *sngA* increased pimaricin production by 1.7 fold, whereas deletion of *sngR* advanced pimaricin production by 6 h and increased its production by 4.6 fold. In *S. chattanoogensis*, another pimaricin-producing strain, *scgA* and *scgX* encode proteins involved in GBL biosynthesis, while *scgR* and its homologous gene *sprA* encode GBL receptor proteins. ScgR and SprA both bind to the promoter of *scgA* and repress its transcription, and SprA also directly represses transcription the *scgR*. Deletion of *scgA*, *scgX*, or *sprA* resulted in morphological differentiation defects and reduced pimaricin production [37].

### 5.3. TetR Family Regulators Modulate Precursor Synthesis

The TetR family is one of the few regulators that can affect both primary and secondary metabolism by regulating acetyl-CoA carboxylase (ACC), the gene for the synthesis of acyl-CoA precursors. In the PKS pathway, acetyl-CoA, malonyl-CoA, and methylmalonyl-CoA are essential as the precursor basic units of the synthetic skeleton, and such regulation is particularly important [38]. Liu et al. found that biosynthesis of avermectin requires a large supply of acyl-CoA, including isobutyryl-CoA, 2-methylbutyryl-CoA, malonyl-CoA, and methylmalonyl-CoA. SAV7472-SAV7473 may be related to the metabolism of pantothenic acid and acyl-CoA. The TetR family protein SAV7471 can inhibit the transcription of *sav7472*-*sav7473* operon directly. At least two other genes involved in acyl-CoA metabolism (SAV1104 and SAV1258) were negatively regulated by SAV7471. SAV7471 may indirectly regulate the biosynthesis of avermectin by negatively regulating the transcription of target genes involved in acyl-CoA metabolism such as SAV7472-SAV7473 [39]. The other TetR family protein (SAV151) negatively regulated avermectin biosynthesis by directly inhibiting the neighboring gene cluster *sav152*-*sav153*-*sav154*. The dehydrogenases and hydrolases encoded by *sav152* and *sav154*, respectively, may provide energy or precursors, thereby indirectly regulating avermectin biosynthesis [40].

**Figure 9:**
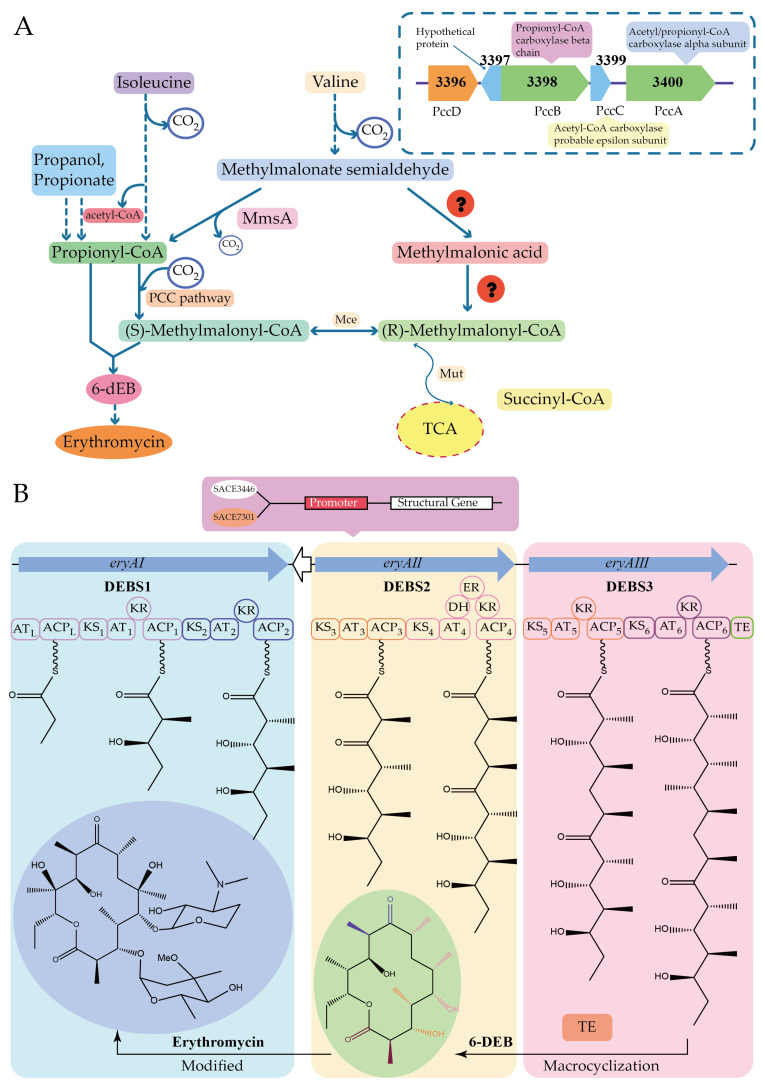
The biosynthesis pathway and gene cluster of erythromycin. (**A**) Effect of PccD on the biosynthesis of erythromycin by regulating *SACE_3398-3400*. (**B**) Effect of SACE_7301 and SACE_3446 on the biosynthesis of erythromycin by regulating *eryAI*.

Xu et al. stimulated erythromycin biosynthesis by increasing the supply of propionyl-CoA precursor, the starting unit of erythromycin, in *Saccharopolyspora erythraea*. Propionyl-CoA is converted to methylmalonyl-CoA by propionyl-CoA carboxylase and is an extended unit of erythromycin. Two malonyl-CoA conversion pathways have been identified in most microorganisms, the methylcitrate cycle (MCC), which converts malonyl-CoA to pyruvate, and the propionyl-CoA carboxylase (PCC) pathway, which is responsible for the metabolism of malonyl-CoA to methylmalonyl-CoA. The addition of n-propanol or propionic acid resulted in a 4- to 16-fold increase in the transcript level of the locus *SACE_3398-3400*, encoding malonyl-CoA carboxylase. PccD, a TetR-family protein, regulates the malonyl-CoA carboxylase encoding gene *SACE_3398-3400* negatively by binding to the t/aTGACGg/cTGt/cTGt/a sequence in its promoter region, which affects erythromycin production by influencing the synthesis of the precursor methylmalonyl-CoA. Deletion of *pccD* resulted in a 15- to 37-fold increase in the transcription level of the *SACE_3398-3400* and an upregulation of propionyl-CoA carboxylase, which converts propanol to propionic acid and is subsequently metabolized to propionyl-phosphate or propionyl-CoA by propionate kinase or acetyl- (propionyl-) CoA synthetase [41] (Figure 9A).

Quantitative analysis of metabolic carbon fluxes indicated that the high consumption rate of propanol increased the intracellular concentrations of propionyl-CoA and methylmalonyl-CoA. And methylmalonic acid, the metabolite of propanol, could act as a ligand of PccD to relieve its negative regulatory effect, which would promote the biosynthesis of erythromycin. Wu et al. found that two TetR family proteins (SACE_7301 and SACE_3446) regulate positively erythromycin biosynthesis by regulating precursor assembly through binding to the promoter of the PKS gene *eryAI* in the erythromycin biosynthesis gene cluster and regulate erythromycin biosynthesis negatively by binding to the promoter region of the resistance gene *ermE* [42,43] (Figure 9B).

The TetR family protein FasR affects primary metabolism by negatively regulating the expression of *accD1*, the genes encoding acetyl-CoA carboxylase, *fasA* and *fasB*, fatty acid synthesis genes, through binding to the AaaANATGAcNaNNtCCTCAtNttT sequence in the promoter region of the target genes in *Corynebacterium glutamicum* [44]. The TetR family protein GdmRIII regulates geldanamycin biosynthesis positively by affecting the transcription of the precursor synthesis gene *gdmH*, and regulates geldanamycin biosynthesis negatively in *S. autolyticus* [45,46]. AccR, a TetR family transcriptional repressor protein, coordinates the short-chain acyl-CoA synthesis pathway in *Streptomyces*. It represses transcription of the *accD1A1*-*hmgL*-*fadE4*, *echA8*, *echA9*, and *fadE2* by binding to a 16-nucleotide palindrome-binding motif (GTTAA-N6-TTAAC) in the promoter region. Deletion of *accR* leads to an increase in the concentration of short-chain acyl-CoA (acetyl-, malonyl-, malonyl-, and methylmalonyl-CoA) that leads to increased avermectin production [47]. The effects of the TetR family of regulators on the synthesis of acyl-CoA-like carbon-flow precursors in the PKS pathway are mainly negatively regulated.

*S. pristinaespiralis* also utilized CoA-like precursors, and the TetR family of regulator (encoded by SSDG_03033) was identified as playing an active role in primithromycin I biosynthesis. Its homologue, PaaR, from *Corynebacterium glutamicum* acts as a transcriptional repressor of the *paa* involved in the catabolism of phenylacetic acid (PAA). L-phenylglycine is a pristinamycin I biosynthesis precursor, which can be generated from phenylacetyl-CoA. PaaR promotes the biosynthesis of pristinamycin I by inhibiting the expression of genes in the *paa* cluster, which allows more phenylacetyl-CoA to flow to the L-phenylglycine biosynthetic pathway [48].

**Figure 10:**
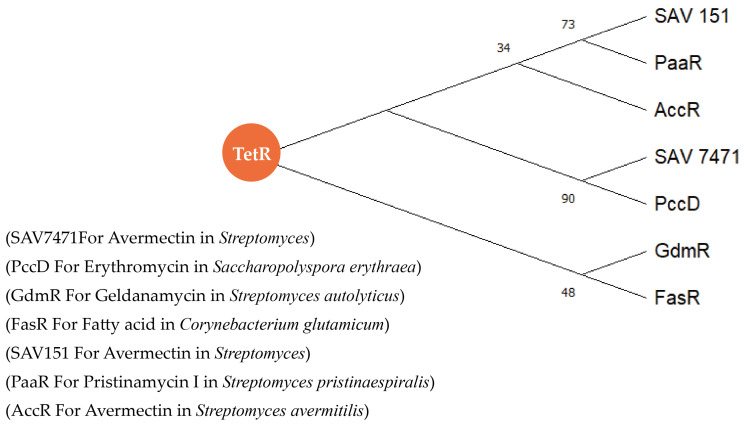
Phylogenetic evolutionary tree of TetR family proteins with the regulatory effect in the biosynthesis precursors.

The amino acid sequences of TetR family proteins in Table 2 were used to construct an evolutionary tree, and the Neighbor-Joining algorithm bootstrap was used to sample the tree 1000 times to increase the confidence of the evolutionary tree. The phylogenetic tree in Figure 10 shows all the reported TetR family regulatory proteins regulating acetyl CoA-like precursors, and their regulatory effects on secondary metabolic pathways are mostly negative. By comparing the binding site sequences of several regulatory proteins with the promoter regions of target genes, except for GdmRIII, it was found that the binding site sequences of TetR family regulators were palindromic sequences. This indicates that we can analyze the potential target genes, precursors synthesis genes, of TetR family proteins by screening the palindromic sequence at the bioinformatics level. This provides us with a better understanding of the functions of TetR family regulatory proteins and the biosynthesis of type I PKS and enables us to explore the secondary metabolic regulation mechanism through precursor regulation.

## 6. New Approach in Precursor Research

In addition to the traditional research methods of precursor biosynthesis and regulation mentioned above, new research methods have been developed. Hong et al. found that rapid and visualized intracellular measurement of malonyl-CoA could be achieved by constructing biosensor for malonyl-CoA in *E. coli*. To compare the concentration changes in malonyl-CoA, seven different malonyl-CoA biosensors were constructed based on the J23119 promoter and realized rapidly and visual measurement of malonyl-CoA by measuring the red fluorescence value. Then, the CRISPR interference (CRISPRi)/*ddCpfl* gene interference system was constructed, and the gene silencing of the target gene was successfully achieved. The effects of different *ddCpfl* mutants and different cRNA targeting positions on gene silencing were compared. Subsequently, attempts were made to optimize the CRISPRi/*ddCpfl* system by fusion of Gp2 and ddCpfl effectors, but the efficiency of gene transcription downregulation was not significantly improved. Finally, CRISPRi/*ddCpfl* technology was used on two target genes (*adhE*/*fabF* and *fabB*, *sucC*) simultaneously, which significantly promoted the accumulation of malonyl-CoA, providing an effective reference for the efficient synthesis of its subsequent target products [49]. Ji et al. utilized the CRISPRi method to achieve a nontoxic conversion of carbon flux. DCpfl was used to replace chemical inhibitors for downregulation of *fabI* gene expression in *E. coli*, and increased the yield of precursor substance butenoic acid by 6 fold (1.41 g/L) [50]. Li et al. applied the CRISPR/*dCpfl* system in *Corynebacterium glutamicum*, which successfully repressed two fluorescent reporter genes simultaneously by expressing *dCpf1* (E1006A, D917A) and a designed single crRNA array. To demonstrate the application of this CRISPR/*dCpf1* system in lysine metabolism, quantitative PCR showed that the system suppressed the transcript levels of four genes involved in lysine biosynthesis (*gltA*, *pck*, *pgi*, and *hom*) by up to more than 90% with a single array and was able to increase lysine production by more than 4 fold [51]. The ability to effectively increase the content of malonyl-CoA precursors in *E. coli* suggests that it is possible to achieve in the secondary metabolism of *Actinomycetes*. This shows that CRISPRi can regulate the transcript levels of precursor synthesis-related genes and thus secondary metabolism biosynthesis, which provides a new idea for the development of novel drugs and the improvement of drug yield.

In addition to altering the biosynthetic pathway to increase product yield as mentioned in this paper, new biosynthetic pathways can be created to aid in the efficient synthesis of antibiotics. In natural evolution, the natural synthetic pathway adopts the “decarboxylation-then carboxylation” pathway of “pyruvate (C3)-acetyl-CoA (C2)-malonyl-CoA (C3)”, which has a series of inherent drawbacks, such as slow catalytic rate, wastage of ATP energy, and strict regulation. Li et al. designed a new pathway “pyruvate (C3)-3-oxopropanoic acid (C3)-malonyl-CoA” (NCM pathway), which realized the biosynthesis of non-acetyl-CoA-dependent malonyl-CoA for the first time, and the catalytic rate of the pathway was 1000-fold higher than that of the natural synthetic pathway. When the NCM pathway was applied to the biosynthesis of fatty acids and polyketides, it was able to achieve a high level of efficiency. Among them, the introduction of this pathway into *Saccharopolyspora spinosa* resulted in an increase in the production of spinosad from 3 g/L to 4.6 g/L, which is the highest production level reported so far, and domestic industrial mass production has been achieved [52]. Through the understanding of various new methods, the experimental ideas and possibilities can be broadened for the development of novel drugs.

## 7. Conclusions

*Actinomycetes* are considered microorganisms which produce natural secondary metabolites in large quantities. Nowadays, researchers mainly analyze the secondary metabolic pathways of different *Actinomycetes* by single-gene function study, transcriptomics and other methods. At the same time, some genetic engineering methods are used to directly manipulate the secondary metabolic biosynthetic gene clusters or to change the copy number of the regulatory gene to expand the yield of target products. The characterization of the growth cycle and secondary metabolic regulatory network of *Actinomycetes* is gradually being refined. Secondary metabolic pathways are closely related to catabolism, undirected metabolism and constitutive metabolism of primary metabolism, and their precursors are usually intermediates of normal or modified primary metabolism. Both chemical precursors and biological precursors in the process of stimulating synthesis can regulate the secondary metabolic biosynthesis process. Based on the characteristics of the precursors, their metabolic pathways can be understood in depth, and increasing the precursors was an effective means to enhance the level of secondary metabolic synthesis. Therefore, innovative approaches can be developed, not only for genetic engineering but also for creating novel synthesis methods and pathways. The greater understanding of precursors means more opportunities to explore new research avenues. The application of the precursor supply system and the dynamic regulatory network of antibiotic biosynthesis in the efficient manufacturing of microbial drugs will be one of the research hotspots in the future.

## Figures and Tables

**Table 1:** Types of precursors required for secondary metabolism ^a^.

Chemical Compound	Precursor
short-chain fatty acid	acetic acid, propionic acid, malonic acid, methylmalonic acid, butyric acid
isoprene	isopentenyl pyrophosphate
amino acids and aromatic intermediates	protein amino acid
sugar and amino sugar	butylose, hexose, pentose, aminosugar
cyclohexanol and aminocyclohexanol	actinomycin, inositol, inosine, streptoguanidine
amidinopyridine	argnine
purine and pyrimidine bases	adenine, guanine, cytosine
aromatic intermediates and aromatic amino acids	mangiferolic acid, branching acid, tyrosine, tryptophan
methyl group	S-adenosylmethionine

^a^ This table summarizes the types and origins of all precursors.

**Table 2:** TetR family regulators modulate precursor synthesis.

Precursor	Product	Regulator	Microorganisms	Binding Site Sequences ^a^	Reference
acyl-CoA	avermectin	SAV7471	*S.avermitilis*	5′-GAGAACSWWCGTTCTC-3′	[39]
–	avermectin	SAV151	*S.avermitilis*	5′-GAACTGACACTCTAGCTTGTCAGTTC-3′ 5′-TCTGACATGGTGTC GTGTCAGA-3′	[40]
propionyl-CoA	erythromycin	PccD	*Saccharopolyspora erythraea*	5′-T/ATGACGG/CTGT/CTGT/A-3′	[41]
acetyl-CoA carboxylase	fatty acids	FasR	*Corynebacterium glutamic*	5′-AAAANATGACNANNTCCTCATNTTT-3′	[44]
malonyl-CoA methoxylmalonyl-CoA	geldanamycin elaiophylin	GdmRIII	*S* *. autolyticus*	5′-CACCATGATGGAGGACCACT-3′ 5′-AGGCCATCGAGGACTGGCTG-3′	[45,46]
isobutyryl-CoA	avermectin	AccR	*S.avermitilis* A14	5′-GTTAA–N_6_–TTAAC-3′	[47]
L-phenylglycine	pristinamycin I	PaaR	*S. pristinaespiralis*	5′-AACGA-N_4_-TCGGT-3′ ^b^	[48]

^a^ S = G or C; W = A or T; N = A, T, C, or G; ^b^ putative binding sites of PaaR.

## Data Availability

Not applicable.
